# Comparison of pediatric poisoning patterns before and during the COVID-19 pandemic in South Korea

**DOI:** 10.1371/journal.pone.0309016

**Published:** 2024-08-16

**Authors:** Juho An, Yura Ko, Heewon Yang

**Affiliations:** Department of Emergency Medicine, Ajou University School of Medicine, Suwon, South Korea; Calcutta National Medical College, Government of West Bengal, INDIA

## Abstract

**Objective:**

To investigate the epidemiological changes in emergency department (ED), including changes in toxic substances and ED outcomes in pediatric and adolescent patients who visited the EDs before and during the COVID-19 pandemic.

**Methods:**

This cross-sectional observational study used data from the ED-based Injury In-depth Surveillance from 2017 to 2021 in South Korea (SK). The study population comprised patients aged <19 years who visited 23 EDs because of poisoning before and during the COVID-19 outbreak. We divided the study period into pre-COVID-19 (January 2017 to February 2020) and COVID-19 periods (March 2020 to December 2021).

**Results:**

In total, 5862 patients were included in the final analysis, with 3863 and 1999 in the pre-COVID-19 and COVID-19 periods, respectively. The patients’ mean age increased from 8.3 ± 7.1 to 11.2 ± 6.9 years between the pre-COVID-19 and COVID-19 periods (P < 0.001), and the number of adolescents (aged 13–18 years) significantly increased during the COVID-19 period (1653 [42.8%] vs. 1252 [62.6%]; P < 0.001). The number of intentional poisoning cases increased from 1332 (34.5%) before COVID-19 to 1174 (58.7%) during COVID-19 (P < 0.001). Specifically, pharmaceutical poisoning significantly increased during the COVID-19 period (2242 [58.0%] vs. 1443 [72.2%]; P < 0.001), with central nervous system (CNS) drug poisoning being the most common type (780 [34.8%] vs. 747 [51.8%]; P < 0.001). Among the intentional poisoning cases, pharmaceutical substance use significantly increased during the COVID-19 period (1207 [90.6%] vs. 1102 [93.9%]; P = 0.007). We used Bayesian structural time series (BSTS) forecasting models to forecast the number of ED visits during COVID-19. The total number of pediatric patients with poisoning decreased during the COVID-19 pandemic. However, when using the BSTS forecasting model, the decrease in the number of patients was not significant. Furthermore, the forecasting models showed no statistically significant increase in the number of intentional pediatric poisoning cases.

**Conclusions:**

The previous studies suggested a decrease in the total number of patients with poisoning but an increase in intentional poisoning cases during the COVID-19 pandemic. By using similar methods to those of previous studies, our results also reached the same conclusion. However, the BSTS model, which predicts real-world time series patterns, seasonal effects, and cumulative effects, shows no significant change in pediatric poisoning patterns during the COVID-19 pandemic.

## 1. Introduction

Pediatric poisoning is a considerable problem, with thousands of children being treated every year and a substantial number of deaths resulting from poisoning [1, 2]. Consequently, public health-related interest in childhood poisoning is increasing [1]. The pattern of childhood poisoning is characterized by two toxicity peaks. Most younger children’s exposure is unintentional (99% of exposures by children aged <6 years), whereas teenage exposure (62% of exposures by children aged 13–19 years) is intentional [3]. In infants, most poisoning cases result from accidental ingestion of household items, such as cleaning products, medicines, and personal care products [4]. These items are often left within reach of a child and can be easily ingested. Accidental ingestion of hazardous substances is the leading cause of injury-related deaths in children aged <5 years [5, 6]. In adolescents, most poisoning cases are due to intentional self-harm, most commonly through the ingestion of medications. The rate of adolescent patients with intentional poisoning has been increasing annually, and these patients may also be at risk of toxicity from illegal drugs and alcohol [4, 7–9].

On March 11, 2020, the World Health Organization declared the COVID-19 outbreak a pandemic, which led to provinces across South Korea (SK) implementing various public health measures, e.g., stay-at-home orders, curfews, and lockdowns to control the spread of severe acute respiratory syndrome coronavirus 2 (SARS-CoV-2) infection. With this policy, the number of patients visiting emergency departments (EDs) has decreased since the COVID-19 outbreak. In the United States, national syndromic surveillance data reported that ED visits decreased by 42% between April 2019 and April 2020 [2]. Although pediatric ED visits decreased significantly during the COVID-19 pandemic, injury-, poisoning-, and mental health-related visits accounted for a larger percentage of all ED visits [10]. Among pediatric patients with poisoning, there has been a significant increase in the number of children accidentally ingesting household chemicals and medications, likely because of the increased time spent at home during the pandemic [11, 12]. Another study showed that intentional poisoning increased during the COVID-19 pandemic in adolescents [8, 12]. The COVID-19 pandemic has brought with it changes in daily routines, increased stress levels, and changes in access to healthcare, all of which have contributed to an increase in pediatric poisoning [12, 13].

To the best of our knowledge, there was no multi-center EDs based study of epidemiological changes in pediatric patients after the ingestion of toxic substances in SK before and during the COVID-19 pandemic. Therefore, this study aimed to investigate changes in the epidemiology, including changes in toxic substances and ED outcomes of poisoning aged <19 years who visited the ED before and during the COVID-19 pandemic. By examining these aspects, we aim to provide a comprehensive understanding of how the pandemic has influenced pediatric poisoning patterns, the types of substances involved, the severity of cases, and the subsequent treatment approaches and outcomes in the emergency setting.

## 2. Materials and methods

### 2.1. Ethics statement

This study was approved by the author’s hospital institutional review board (AJOUIRB-DB-2022-530).

### 2.2. Study design and setting

This retrospective multicenter observational study was performed using the Korean Emergency Department-Based Injury In-Depth Surveillance (EDIIS) database. This database is a nationwide, 23-center, ED-based injury registry conducted by the Korean Centers for Disease Control and Prevention. The EDIIS collects data that aid in developing a national policy for injury prevention and performs periodic quality control by analyzing errors.

### 2.3. Study population and data collection

The study population comprised patients aged <19 years who visited 23 EDs because of poisoning before and during the COVID-19 outbreak. We divided the study period into pre-COVID-19 (January 2017 to February 2020) and COVID-19 periods (March 2020 to December 2021). The mechanism of injury variable of “intoxication” was extracted and reviewed from the EDIIS database. Among patients with intoxication, those who had substance exposure to skin or eyes and those with insufficient medical records were excluded from the analysis. Patients who were transferred from other hospitals were also included in the study.

Basal characteristics included age, sex, place of poisoning, ED visit tool, and intentionality of poisoning. We divided the poisonous substances into three groups: pharmaceuticals, non-pharmaceuticals, and gases [14]. ED outcomes included hospitalization and ED length of stay (EDLOS).

### 2.4. Statistical analyses

Data are presented as the mean ± standard deviation, median (interquartile range) for continuous variables, and number (%) for categorical variables. To compare the characteristics and proportion of patients visiting EDs for poisoning before and after the COVID-19 pandemic, we used an independent sample t-test to analyze continuous variables. Categorical variables were analyzed using the chi-square and Fisher exact tests. Statistical analyses were performed using R software (version 4.3.0, The R Foundation for Statistical Computing, Vienna, Austria). Statistical significance was set at P < 0.05.

We also used Bayesian structural time series (BSTS) forecasting models to estimate the forecasted number of patients with poisoning during the COVID-19 period and compared them with the actual number of patients to determine whether the characteristics of pediatric toxicity changed because of the COVID-19 outbreak. Our analysis also incorporated an examination of cumulative effects, assessing how the extended duration of the pandemic influenced trends in pediatric poisonings over time.

Lastly, the forecasted number of patients with poisoning during the COVID-19 period (2021) was estimated based on models trained using pre-COVID-19 data (2017–2019). These forecasted numbers represented the degree of pediatric toxicity, assuming that the COVID-19 pandemic did not occur. This metric was used to assess the impact of the COVID-19 pandemic.

## 3. Results

### 3.1. General characteristics of patients with poisoning between the pre-COVID-19 and COVID-19 periods

In total, 5862 patients were included in the final analysis, comprising 3863 and 1999 in the pre-COVID-19 and COVID-19 periods, respectively (Table 1). Approximately 101.7 and 90.0 patients had poisoning per month in the pre-COVID-19 and COVID-19 periods, respectively. The patients’ mean age increased significantly from 8.3 ± 7.1 to 11.2 ± 6.9 years between the pre-COVID-19 and COVID-19 periods (P < 0.001). Notably, the proportion of adolescent patients (age 13–18 years) significantly increased during the COVID-19 period (1653 [42.8%] vs. 1252 [62.6%]; P < 0.001).

**Table 1:** General characteristics.

	Pre-COVID-19 (n = 3863)	COVID-19 (n = 1999)	P value
Number/month	101.7	90.0	
Age	8.3 (7.1)	11.2 (6.9)	<0.001
Age group			<0.001
0–5	1918 (49.65)	632 (31.62)	
6–12	292 (7.56)	115 (5.75)	
13–18	1653 (42.79)	1252 (62.63)	
Gender			<0.001
Male	1613(41.76)	642(32.12)	
Female	2250 (58.24)	1357 (67.88)	
Place			<0.001
Home	3389 (87.73)	1829 (91.5)	
Others	474 (12.27)	170 (8.5)	
ED visit tools			<0.001
Ambulance	1067 (27.62)	681 (34.07)	
Self-visit	2796 (72.38)	1318 (65.93)	
Intentionality			
Yes	1332 (34.48)	1174 (58.73)	<0.001
No	2531(65.52)	825(41.27)	
Pharmaceutical, n (%)	2242 (58.04)	1443 (72.19)	<0.001
CNS drug	780 (34.79)	747 (51.77)	
Analgesics/antipyretics	652 (29.08)	428 (29.66)	
URI drug*	306 (13.65)	70 (4.85)	
Hormones/Vitamin	184 (8.21)	70 (4.85)	
Cardiovascular drugs	145 (6.47)	62 (4.3)	
Gastrointestinal	35 (1.56)	20 (1.39)	
Parenteral	55 (2.45)	16 (1.11)	
Others	85 (3.79)	30 (2.08)	
Non-pharmaceutical	1123 (29.07)	424 (21.21)	0.139
Household products	466 (41.5)	163 (38.44)	
Corrosive	107 (9.53)	40 (9.43)	
Pesticide/insecticide	313 (27.87)	129 (30.42)	
Alcohol	71 (6.32)	40 (9.43)	
Others	166 (14.78)	52 (12.26)	
Gas	498 (12.89)	132 (6.6)	<0.001
ED outcome			<0.001
Discharge	2971 (76.91)	1456 (72.84)	
Hospitalization	805 (20.84)	502 (25.11)	
Transfer	85 (2.2)	37 (1.85)	
Death in ED	2 (0.05)	4 (0.2)	
ED LOS (hour)	2.8 [0,67]	3.85 [0,62]	<0.001

Data is presented as mean (standard deviation) and median [range] for the continuous variables and number (%) for the categorical variables

*Upper respiratory infection (URI) drugs, including antitussive, anti-histamines, and antimicrobials

The proportion of intentional poisoning cases increased from 34.5% in the pre-COVID-19 period to 58.7% during the COVID-19 period (P < 0.001). The proportion of pharmaceutical poisoning significantly increased during the COVID-19 period (P < 0.001), with central nervous system (CNS) drug poisoning cases being the most common type. The use of respiratory drugs, antihistamines, and antimicrobial drugs decreased during the COVID-19 period in pharmaceutical poisoning cases. The proportion of non-pharmaceutical poisoning cases statistically significantly decreased during the COVID-19 period.

### 3.2. Intentional poisoning

Approximately 35.1 and 53.4 patients/month had intentional poisoning in the pre-COVID and COVID-19 periods, respectively (Table 2). Adolescent and female patients were predominant in both periods, with home being the most common location of poisoning. The proportion of pharmaceutical poisoning cases increased during the COVID-19 period, and that of CNS drug cases also increased. Regarding ED outcomes, the hospitalization rate decreased in the COVID-19 period.

**Table 2:** Intentional poisoning.

	Pre-COVID-19 (n = 1332)	COVID-19 (n = 1174)	P value
Number/month	35.1	53.4	
Age group			0.477
0–5	4 (0.3)	1 (0.09)	
6–12	28 (2.1)	28 (2.39)	
13–18	1300 (97.6)	1145 (97.53)	
Gender			0.096
Male	266 (19.97)	203 (17.29)	
Female	1066 (80.03)	971 (82.71)	
Place			0.034
Home	1166 (87.54)	1060 (90.29)	
Others	166 (12.46)	114 (9.71)	
Pharmaceutical, n (%)	1207 (90.62)	1102 (93.87)	<0.001
CNS drug	639 (52.94)	675 (61.25)	
Analgesics/antipyretics	481 (39.85)	373 (33.85)	
URI drug*	31 (2.57)	15 (1.36)	
Hormones/ Vitamin	13 (1.08)	2 (0.18)	
Cardiovascular drugs	29 (2.4)	20 (1.81)	
Gastrointestinal	4 (0.33)	9 (0.82)	
Others	10 (0.83)	8 (0.73)	
Non-pharmaceutical	78 (5.86)	50 (4.26)	0.904
Household products	27 (34.62)	16 (32)	
Corrosive	5 (6.41)	4 (8)	
Pesticide/insecticide	36 (46.15)	21 (42)	
Alcohol	7 (8.97)	7 (14)	
Others	3 (3.85)	2 (4)	
Gas	47 (3.53)	22 (1.87)	0.016
ED outcome			<0.001
Discharge	733 (55.03)	732 (62.35)	
Hospitalization	532 (39.94)	412 (35.09)	
Transfer	65 (4.88)	29 (2.47)	
Death in ED	2 (0.15)	1 (0.09)	
ED LOS (hour)	8.2 ± 7.4	8.5 ± 7.2	0.455

Data is presented as mean (standard deviation) for the continuous variables and number (%) for the categorical variables

*Upper respiratory infection (URI) drugs, including antitussive, anti-histamines, and antimicrobials

### 3.3. Accidental poisoning

Accidental poisoning occurred in approximately 66.6 and 37.5 patients per month in the pre-COVID-19 and COVID-19 periods, respectively. Most poisoning cases occurred in younger age groups, and male patients were predominant in both periods. The most common place of poisoning was the home (Table 3). Regarding pharmaceutical poisoning cases, the proportion of CNS poisoning cases increased during the COVID-19 period, whereas that of respiratory drug cases decreased.

**Table 3:** Accidental poisoning.

	Pre-COVID-19 (n = 2531)	COVID-19 (n = 825)	P value
Number/month	66.6	37.5	
Age group			0.778
0–5	1914 (75.62)	631 (76.48)	
6–12	264 (10.43)	87 (10.55)	
13–18	353 (13.95)	107 (12.97)	
Gender			>0.999
Male	1347 (53.32)	439 (53.21)	
Female	1184 (46.78)	386 (46.79)	
Place			<0.001
Home	2223 (87.83)	769 (93.21)	
Others	308 (12.17)	56 (6.79)	
Pharmaceutical, n (%)	1035 (40.89)	341 (41.33)	0.001
CNS drugs	141 (13.62)	72 (21.11)	
Analgesics/Antipyretics	171 (16.52)	55 (16.13)	
URI drug*	275 (26.57)	55 (16.13)	
Hormones/ Vitamin	171 (16.52)	68 (19.94)	
Cardiovascular drugs	116 (11.21)	42 (12.32)	
Gastrointestinal	31 (3)	11 (3.23)	
Others	130 (12.56)	38 (11.14)	
Non-pharmaceutical	1045 (41.29)	374 (45.33)	0.297
Household products	439 (42.01)	147 (39.3)	
Corrosive	102 (9.76)	36 (9.63)	
Pesticide/insecticide	277 (26.51)	108 (28.88)	
Alcohol	64 (6.12)	33 (8.82)	
Others	163 (15.6)	50 (13.37)	
Gas	451 (17.82)	110 (13.33)	0.003
ED outcome			0.044
Discharge	2238 (88.42)	724 (87.76)	
Hospitalization	273 (10.79)	90 (10.91)	
Transfer	20 (0.79)	8 (0.97)	
Death in ED	0 (0)	3 (0.36)	
ED LOS (hour)	3.0 ± 2.9	3.3 ± 3.1	0.002

Data is presented as mean (standard deviation) for the continuous variables and number (%) for the categorical variables

*Upper respiratory infection (URI) drugs, including antitussive, anti-histamines, and antimicrobials

### 3.4. BSTS forecasting models

**Fig 1:**
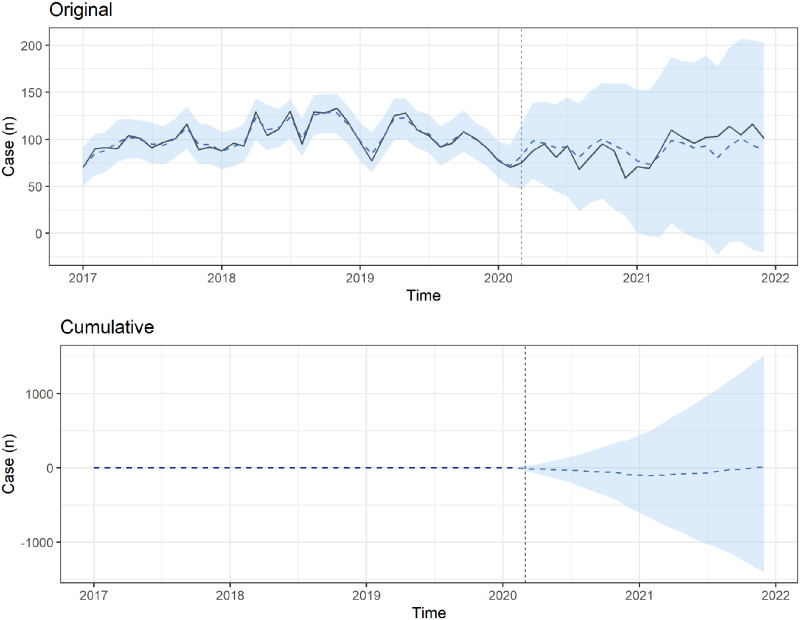
Bayesian structural time series models estimating the observed (solid line) and forecasted (dashed line) numbers of total patients with poisoning who visited emergency departments during the COVID-19 pandemic. The cumulative differences between forecasted and observed numbers of patients with poisoning who visited emergency departments in the COVID-19 period.

**Fig 2:**
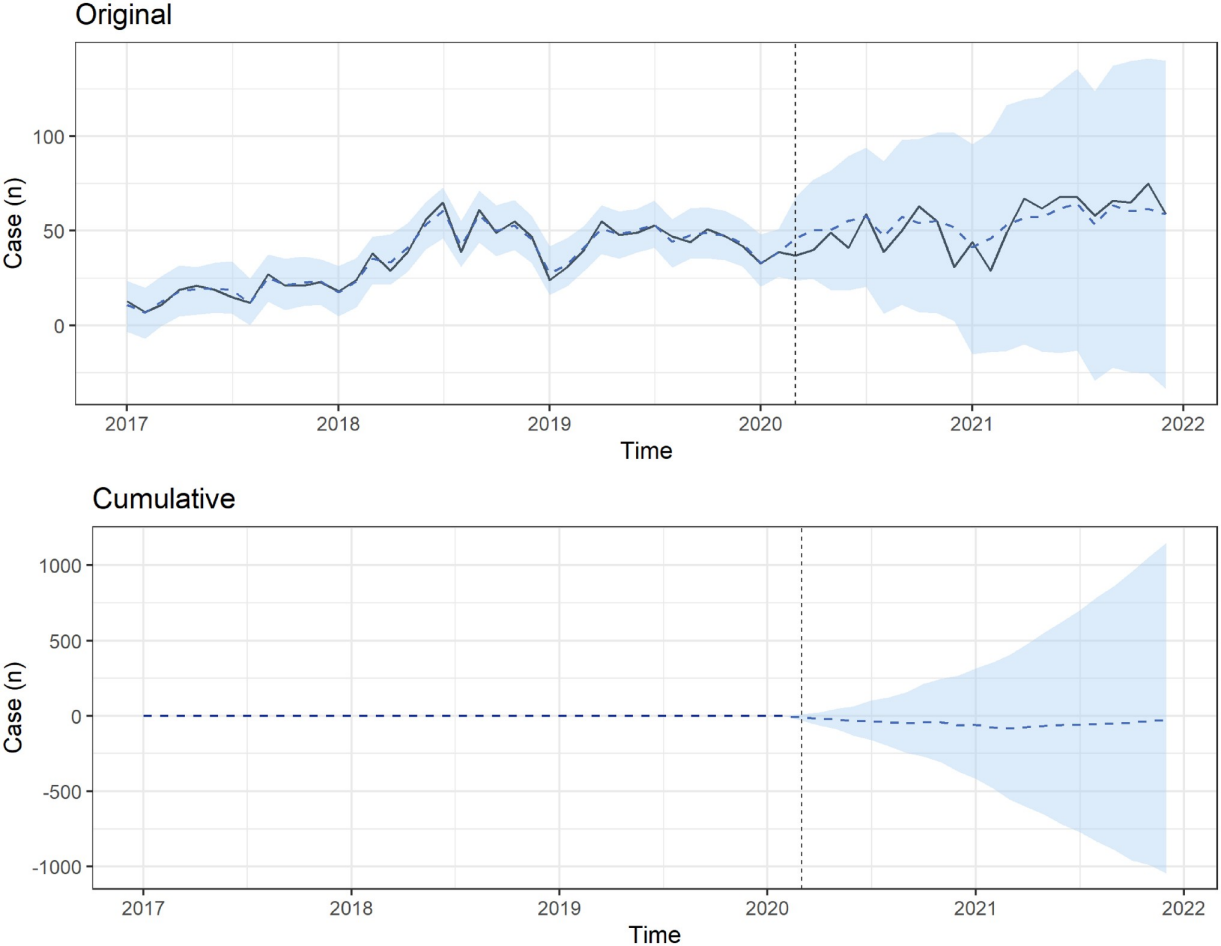
Bayesian structural time series models estimating the observed (solid line) and forecasted (dashed line) numbers of patients with intentional poisoning who visited emergency departments during the COVID-19 pandemic. The cumulative differences between forecasted and observed numbers of patients with poisoning who visited emergency departments in the COVID-19 period.

**Fig 3:**
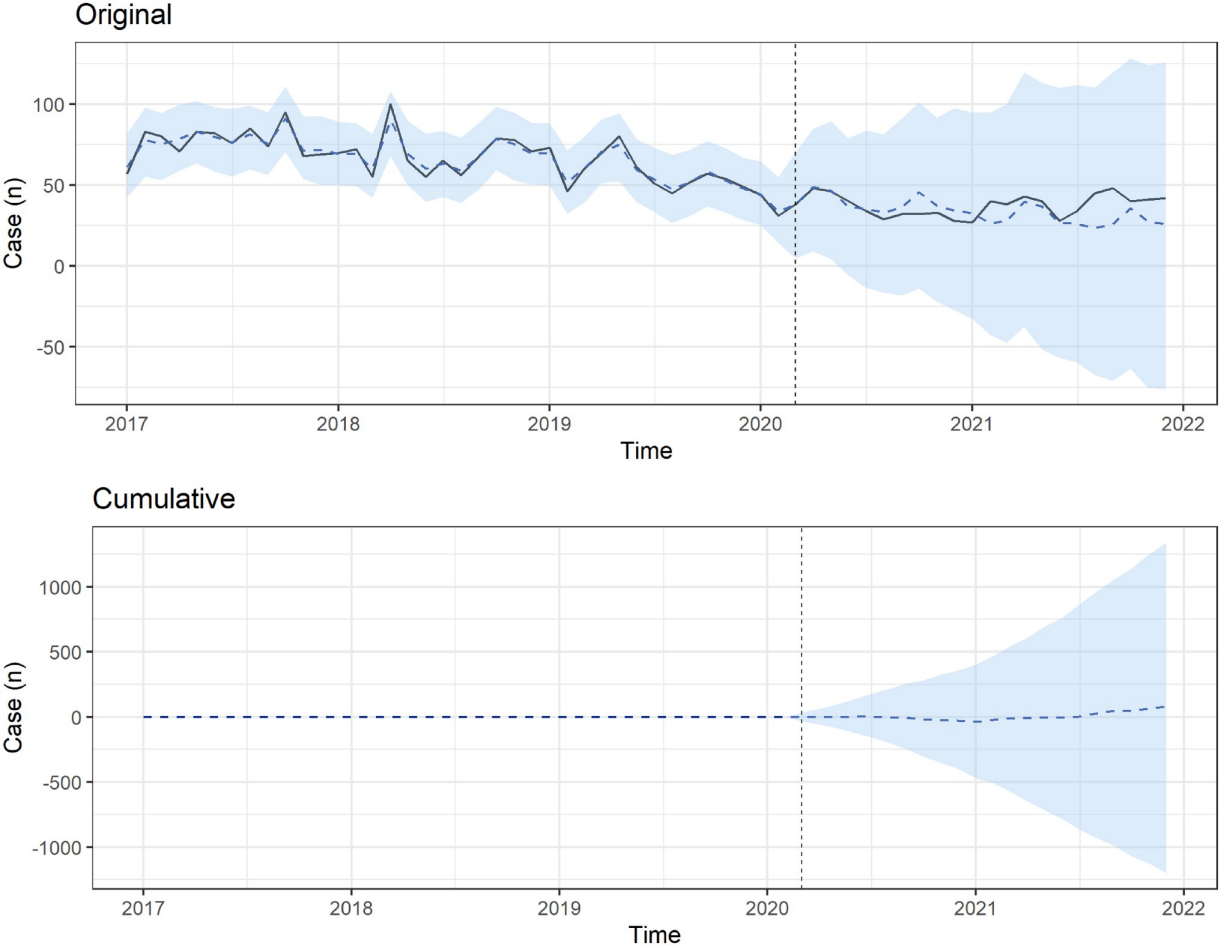
Bayesian structural time series models estimating the observed (solid line) and forecasted (dashed line) numbers of patients with accidental poisoning who visited emergency departments during the COVID-19 pandemic. The cumulative differences between forecasted and observed numbers of patients with poisoning who visited emergency departments in the COVID-19 period.

We used BSTS forecasting models to estimate the forecasted number of ED visits during the COVID-19 period (Fig 1). Compared with pre-COVID-19 periods, the total number of pediatric patients with poisoning decreased during the COVID-19 period. However, more pediatric patients with poisoning visited the ED approximately 2 years after the COVID-19 outbreak (December 2021) without statistical significance (Fig 1). To compare intentional and accidental poisoning, we also used BSTS forecasting models (Figs 2 and 3). Both intentional and accidental forecasting models showed no statistically significant increase in the number of pediatric patients with poisoning. The cumulative difference analysis revealed that the actual number of pediatric poisoning incidents did not significantly exceed the forecasted number (Figs 1–3).

## 4. Discussion

Our multicenter registry-based study showed an increase in the rate of intentional and adolescent poisoning during the COVID-19 pandemic. However, the BSTS forecasting model showed no differences in the number of patients who visited the 23 EDs in SK. This is the first study to estimate the forecasted number of poisoning cases in pediatric and adolescent patients during the COVID-19 pandemic and to compare that number with the actual number of patients to determine whether the characteristics of pediatric toxicity have changed due to COVID-19.

In the present study, there was a significant increase in the mean age from 8.3 ± 7.1 to 11.2 ± 6.9 years between the pre-COVID-19 and COVID-19 periods (P < 0.001). This trend may be due to the increased proportion of adolescent patients. Similar findings were observed in a Canadian study, where significant changes in age distribution were noted, with an increased proportion of teenagers (aged 11–18 years) [12]. In a single-center study conducted in SK, the mean age increased from 7 to 10 years during the COVID-19 period and was accompanied by an increase in the proportion of patients aged >10 years [8]. Furthermore, a previous study reported an increase in the proportion of teenagers in the USA. [15].

Moreover, our study showed that the proportion of intentional poisoning cases increased significantly during the COVID-19 pandemic. Strong policies, e.g., stay-at-home orders, curfews, and lockdowns, to control the spread of SARS-CoV-2 infection have a negative impact on pediatric mental health. There has been an increase in pediatric hospital presentations related to deliberate self-harm behaviors [16]. This finding is similar to those of previous studies [8, 15]. Pharmaceutical CNS drugs were the most common agents of intentional poisoning among the pediatric patients in this study.

The home was the most common location of both intentional and accidental poisoning cases. This finding may be attributed to the school lockdowns and stay-at-home policies. Although the proportion of poisoning cases at home increased, the total number of cases and the proportion of accidental poisoning cases decreased. This reduction may be due to the increased parental monitoring and precautions resulting from parental teleworking. Michael et al. also reported no association with healthcare referrals despite an increase in calls for exploratory pediatric ingestion during the COVID-19 pandemic [17].

Interestingly, the rate of poisoning by URI drugs decreased significantly from 481 (39.85%) to 373 (33.85%) in intentional cases and from 275 (26.57%) to 55 (16.13%) in accidental cases. This reduction could be attributed to a significant decrease in the number of visits for respiratory disorders. A study conducted in the United States reported a significant 70% decrease in visits for respiratory disorders during the COVID-19 pandemic [11]. This finding could have been due to the difficulty in accessing the necessary medication supplements during the pandemic, or it could have been caused by the impact the pandemic has had on healthcare-seeking behavior and overall healthcare utilization.

Several previous studies have suggested that the increased ratio of pediatric patients with intentional poisoning is attributable to the policy of contagion isolation and social distancing, which resulted in mental health problems during the COVID-19 pandemic [8, 12, 15]. However, the total number of patients visiting the EDs decreased considerably during the same period, although the proportion of pediatric patients with poisoning may have increased during the pandemic [11]. We analyzed multicenter ED-based data from SK to evaluate the BSTS forecasting model in pediatric patients with poisoning according to suicide and self-harm attempts. The results showed no statistical difference between the forecasting model result and the actual number of pediatric patients with poisoning.

In our study, compared with the BSTS forecasting model result, the actual number of pediatric patients with unintentional poisoning decreased until January 2021. Similarly, the actual number of pediatric patients with intentional poisoning tended to decrease during the same period. For these reasons, our study suggests that the actual number of pediatric patients with poisoning decreased, and there was weak statistical evidence of an increase in the number of pediatric patients who visited EDs with poisoning during the pandemic.

We analyzed five years of pediatric poisoning patterns in SK, with a specific focus on the period during and before the COVID-19 pandemic which is the first analysis by using national EDIIS data in SK. We identified the change of pediatric poisoning patterns; intentional vs. accidental poisoning, category of drugs, other substances. The previous studies concluded that a decrease in the total number of pediatric poisoning patients and an increasing proportion of intentional poisoning [12, 14]. In our study, by analyzing the time series BSTS model, which predicts real-world time series patterns, seasonal effects, and cumulative effects, shows no significant change in the number of pediatric poisoning patients during the COVID-19 pandemic. Additionally, we observed changes in the substances involved in poisonings, notably CNS drugs, particularly among adolescents aged 12–18 years which highlights the importance of addressing youth suicide in Korea. We might suggest that COVID-19 is just one of several social and environmental factors influencing pediatric poisoning patterns in SK, and that the issue of adolescent suicide may not be solely due to COVID-19. By understanding the shift in pediatric poisoning patterns, the society can develop policies to prevent pediatric poisoning in SK.

Our study had several limitations. First, concerning the data from the multicenter registry, ED outcomes may have been partially influenced by the lack of a standardized protocol for hospitalization among the participating EDs. As the current EDIIS study continues and follow-up studies continue to emerge, it will be necessary to develop and implement standardized hospitalization and treatment protocols across all Eds. Second, the study did not account for the possibility of transferring some cases to nearby centers due to the social distance policy of COVID-19 in SK. Furthermore, changes in the admission protocol during the COVID-19 crisis, such as allowing consultation from home through hotline numbers to decrease the number of admitted cases to EDs and reduce the risk of COVID-19 transmission, were not considered. These factors may have influenced the actual number of pediatric poisoning cases reported. Third, our study did not consider the long-term effect of COVID-19 among pediatric patients with poisoning, including the post-pandemic period. Thus, further studies are required to evaluate the long-term effects of COVID-19 on pediatric patients with poisoning, including the post-pandemic period is necessary.

## 5. Conclusions

Our multicenter registry-based study showed a decreasing total number of pediatric patients with poisoning during the COVID-19 pandemic. The BSTS forecasting model showed no differences in the number of patients who visited the 23 EDs in SK. However, the number of patients with intentional poisoning, mean patient age, and intentional CNS drug poisoning cases increased during the COVID-19 pandemic.
